# Analysis of β-Methylphenethylamine (BMPEA) and Its Novel Metabolites in Rat Blood Using MMSPE and UPLC-qTOF-MS

**DOI:** 10.3390/toxics13121011

**Published:** 2025-11-22

**Authors:** Ahmad Alamir, James Watterson, Ibraheem Attafi

**Affiliations:** 1 Forensic Toxicology Services, Ministry of Health Branch in Jazan Region, Jazan 45142, Saudi Arabia; 2 Department of Chemistry and Biochemistry, Laurentian University, Sudbury, ON P3E 2C6, Canada; 3 Department of Forensic Science, Laurentian University, Sudbury, ON P3E 2C6, Canada; jwatterson@laurentian.ca; 4 South Al-Qunfudah General Hospital, Makkah Health Cluster, Makkah 21912, Saudi Arabia

**Keywords:** amphetamine-related substances, forensic toxicology, β-methylphenethylamine (BMPEA), 1-amino-2-phenylpropan-2-ol, UPLC-qTOF-MS

## Abstract

β-Methylphenethylamine (BMPEA), a positional isomer of amphetamine increasingly detected in dietary supplements and weight-loss products, poses significant analytical challenges in forensic and doping control due to its structural similarity to amphetamine. This study presents a validated analytical workflow combining mixed-mode solid-phase extraction (MMSPE) with ultra-performance liquid chromatography–quadrupole time-of-flight mass spectrometry (UPLC-qTOF-MS) for the selective quantification of BMPEA and identification of its metabolites in rat cardiac blood. Blood was taken at 20 and 90 min after injection from twelve adult male Sprague-Dawley rats that were randomly assigned to four groups (*n* = 3): an untreated control, a low-dose cohort (10 mg/kg, i.p.), and two high-dose cohorts (30 mg/kg, i.p.). The technique demonstrated strong differentiation between BMPEA and amphetamine isomers, excellent linearity over 20–1000 ng/mL (R^2^ > 0.99), and quantification limits appropriate for forensic applications. A short biological half-life and quick elimination kinetics are consistent with related phenethylamines, as evidenced by the peak BMPEA concentrations of 899 ng/mL at 20 min and 22 ng/mL at 90 min. Comprehensive low- and high-energy mass spectrometric analyses revealed a novel BMPEA metabolite, characterized as 1-amino-2-phenylpropan-2-ol, based on fragmentation patterns and retention time comparison with reference standards. This work delivers a rigorous, high-sensitivity analytical tool for BMPEA detection in biological matrices and enhances understanding of its metabolic fate, offering critical biomarkers for forensic toxicology and anti-doping investigations.

## 1. Introduction

**Figure 1:**
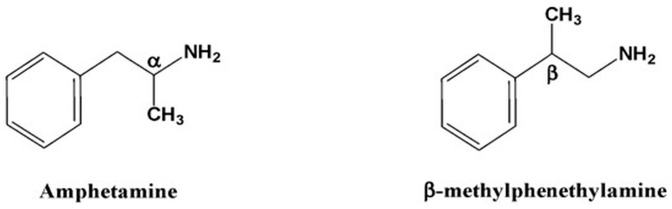
Chemical structures of amphetamine (1-phenylpropan-2-amine); β-Methylphenethylamine, BMPEA (2-phenylpropane-1-amine).

A synthetic compound in the phenethylamine class, β-methylphenethylamine (BMPEA) is a positional isomer of amphetamine that differs in the location of the methyl group but shares a similar core structure. BMPEA was first synthesized in 1930 as a substitute for amphetamine. Since then, it has been found in a number of dietary supplements and weight-loss products, frequently without proper labeling, which has raised questions about its uncontrolled use and abuse potential [1,2,3]. The chemical structures of amphetamine and BMPEA are depicted in Figure 1.

BMPEA is a dopamine and norepinephrine transporter substrate, but its potency at dopamine transporters is about ten times lower than that of amphetamine. Importantly, BMPEA has strong effects on norepinephrine transporters; it has been shown to increase blood pressure (BP) in rats and may cause adverse cardiovascular effects via substrate activity at peripheral norepinephrine sites, while producing minimal stimulant effects on locomotor activity compared to amphetamine [3,4]. These pharmacological distinctions underscore the critical importance of accurate detection and differentiation of BMPEA from amphetamine in forensic and clinical toxicology contexts.

The detection and differentiation of BMPEA from amphetamine present formidable analytical challenges, as both compounds share nearly identical molecular weights and produce remarkably similar mass spectrometric fragmentation patterns. Given that their mass spectrometric profiles are almost identical, the presence of BMPEA in sports supplements has posed significant health risks and unintentional doping violations when BMPEA was misidentified as amphetamine, leading to serious consequences for athletes, underscoring the significance of precise detection and differentiation from amphetamine [5,6,7]. Different isomers differ in terms of safety and efficacy, as well as regulatory controls. Isomeric form identification is therefore becoming increasingly crucial, especially in forensic and doping control applications.

To address these analytical challenges, this study employs a validated ultra-performance liquid chromatography–quadrupole time-of-flight mass spectrometry (UPLC-qTOF-MS) method in conjunction with mixed-mode solid-phase extraction to quantify BMPEA in rat blood with isomeric differentiation and uses MS^E^ fragmentation to detect and characterize its metabolites to elucidate potential biotransformation pathways in rat cardiac blood [7]. This approach advances the toxicological and forensic understanding of BMPEA exposure and metabolism.

## 2. Materials and Methods

### 2.1. Chemicals and Materials

Standards for (±) amphetamine, (S,S)-(+)-pseudoephedrine, (1S,2R)-(+)-ephedrine-d_3_ HCl, and (±)-amphetamine-d_11_ were purchased from Cerilliant (Round Rock, TX, USA) as 1 mg/mL methanolic solutions and diluted as required. (R*,S*)-(±)-ephedrine HCl, DL-norephedrine, and (R)-(+)-β-methylphenethylamine were obtained from Sigma Aldrich (Oakville, ON, Canada) as 1 mg/mL methanolic solutions and diluted as required. (+)-Norpseudoephedrine hydrochloride (cathine hydrochloride) was purchased from LGC Standards (Manchester, NH, USA) as a 0.1 mg/mL methanolic solution and diluted as required. ACN, MeOH, and purified water, used in drug extraction and UPLC analysis, were of reagent grade and obtained from EMD Milipore (Billerica, MA, USA). Ammonium acetate was purchased from Mallinckrodt Baker Inc. (Phillipsburg, NJ, USA). Acetic acid and HCl were obtained from BDH (Radnor, PA, USA). Ammonium hydroxide, ammonium formate, and formic acid were purchased from Fisher Chemicals (Bridgewater, NJ, USA). Amphetamines-specific MIP-SPE (25 mg) was purchased from Biotage (Uppsala, Sweden). Mixed-mode SPE (Oasis MCX, 30 mg) and FTPE (HLB Prime, 100 mg) were purchased from Waters (Milford, MA, USA). Aged animal blood was obtained from Ottawa Laboratory (Nepean, ON, Canada). Blank human whole blood was obtained from Utak Laboratories Inc. (Valencia, CA, USA).

### 2.2. Drug Administration to Rats and Blood Sampling

Male Sprague-Dawley rats (230–250 g, *n* = 12) were provided by Charles River Laboratories (St-Constant, QC, Canada). The animals were housed in an environmentally controlled breeding room at the Laurentian University Animal Care Facility and were acclimated to the laboratory conditions for 3 days. Adult male Sprague-Dawley rats were housed in groups of three in cages with ¼″ bedding (Harlan Teklad, Indianapolis, IN, USA) under a 12 h light/dark cycle at a temperature of 20 °C. The animals were provided with free access to water and Harlan Teklad laboratory diet 8640. The animal procedures used in this study were approved by the Laurentian University Animal Care Committee. Twelve adult male Sprague-Dawley rats were randomly assigned to four groups (*n* = 3 each): a control group, in which the rats did not receive BMPEA; a low-dose group where the rats received a dose of 10 mg/kg of BMPEA (i.p.); and two high-dose groups where rats were injected with a dose of 30 mg/kg (i.p.). The rats from the low-dose and one of the two high-dose groups were euthanized by CO2 asphyxiation within 20 min. Those from the other high-dose group were euthanized by CO2 asphyxiation 90 min post-injection. Perimortem blood samples were obtained from the rat heart (cardiac puncture). The blood was collected in sodium fluoride vacutainer tubes obtained from BD (Mississauga, ON, Canada).

### 2.3. UPLC–qTOF-MS Method

The methodology adopted in this work was initially developed in the Master’s thesis of Ahmad Alamir [8] and is here further refined and verified.

Chromatographic separations (UPLC) were performed using a Waters ACQUITY UPLC™ system (Waters Corporation, Milford, MA, USA) with HSS T3 column (100 mm × 2.1 mm, 1.8 µm) at 45 °C; autosampler at 10 °C; injection volume 5 µL; flow rate at 0.5 mL/min; and total runtime 15 min. Mobile phase A: purified water 0.1% (*v*/*v*) formic acid + 5 mM ammonium formate (pH 2.9). Mobile phase B: acetonitrile + 0.1% (*v*/*v*) formic acid. Chromatographic separation was achieved using a pseudo-isocratic gradient elution program designed to achieve baseline resolution of isomeric amphetamine-related compounds. The gradient profile was programmed as follows: 0–1 min, 0% B (isocratic); 1–10 min, 5% B (pseudo-isocratic for isomer baseline analytes); 10–11 min, 5–30% B; 11–12 min, 30–50% B; 12–13 min, 50–100% B (column washing); 13–14 min, 100–0% B; and 14–15 min, 0% B (re-equilibration).

Mass spectrometric detection was performed using a Waters Xevo G2-XS qTOF with ESI+, StepWave (Waters Corporation, Milford, MA, USA). Ion Source: capillary 0.8 kV, sampling cone 20 V, source 140 °C, desolvation 250 °C, desolvation gas flow 900 L/h (nitrogen), cone gas flow 50 L/h (nitrogen). Acquisition: MSE (centroid mode); *m*/*z* 50–601; 0.1 s per scan; low energy (LE) 4 eV; HE 10–40 eV; resolution >20,000 full width at half maximum (FWHM) at *m*/*z* 278. External reference (LockSpray™): leucine enkephalin (*m*/*z* 556.2771) at 5 µL/min every 5 s; mass accuracy typically ≤5 ppm.

### 2.4. BMPEA Concentration Determination

Two standard curves were constructed using six calibrator samples in duplicate for each analyte. Calibrators were prepared in a drug-free rat cardiac blood matrix (control group). Rat blood calibrators (250 μL) were assayed at concentrations of 20, 40, 200, 500, 800, and 1000 ng/mL using a combined working standard solution, with addition of 125 ng ISs to each calibrant. Quantification was performed based on the ratio of the integrated area under EIC of BMPEA to that of amphetamine-d_11_ using specific quantifier ions (Table 1).

### 2.5. BMPEA Metabolite Identification

The identification process of the BMPEA metabolite was carried out by comparison with drug-free samples. BMPEA metabolites were identified using a combined automated–manual workflow under Scientific Working Group for Forensic Toxicology (SWGTOX)-aligned criteria, requiring multiple orthogonal identifiers. Data were acquired in MSE mode; low- and high-energy spectra from BMPEA-dosed rat blood were compared to drug-free controls to flag ions unique to exposed samples, followed by structural elucidation from HE fragments to exclude endogenous interferences.

Identification required all of the following: (i) retention time within ±0.05 min versus structurally related references (norephedrine, cathine); (ii) mass accuracy within ±10 millidaltons (mDa) (≈5–10 ppm) for protonated molecular ion [M + H]^+^ (precursor) and diagnostic fragment ion at *m*/*z* 134.0960 ± 0.01; (iii) concordant low-energy (LE)/high-energy (HE) fragmentation consistent with the proposed chemical structure, including ions at *m*/*z* 134.0960 (protonated molecular ion after water loss), 117.0716 (subsequent aromatic rearrangement), and 115.0557 (further fragmentation); and (iv) isotope pattern matching to theoretical distributions within acceptable tolerances for compounds containing carbon, hydrogen, nitrogen, and oxygen. Only compounds meeting all four criteria were reported as confidently identified metabolites. Molecular or fragmented ions uniquely found in LE mass spectral profiles of BMPEA-dosed samples were considered potential metabolites and were used for subsequent analysis by examining their HE mass spectral profiles.

### 2.6. Data Processing

The raw data obtained after analysis were processed by two types of software. MassLynx 4.1 software (Waters, Manchester, UK) was employed for manual assessment of analyte identification criteria, enabling detailed evaluation of retention time precision, mass accuracy, and fragmentation pattern consistency. Parallel to manual evaluation, UNIFI 1.7.0 software (Waters, Manchester, UK) provided automated metabolite identification through streamlined workflow processing, utilizing built-in spectral libraries and intelligent peak detection algorithms.

Analyte identification criteria were manually assessed using Masslynx 4.1 software (Waters, Manchester, UK); the raw data were also processed automatically using the streamlined workflow of the UNIFI 1.7.0 software (Waters, Manchester, UK) for identification and quantification of the analytes. Compound identification was based on retention time (±0.05 min), mass deviation (±10 mDa) and appropriate isotope profile.

## 3. Results

The method was validated per SWGTOX, including selectivity and specificity. Triplicate extracts from five drug-free blood matrices (aged bovine, aged sheep, and three human sources) were analyzed by UPLC-qTOF-MS with no endogenous interferences observed. Matrix effects (%ME) ranged from −21% to +9%, and mixed-mode solid-phase extraction (MMSPE) recoveries were 63–90%, both within SWGTOX limits. Carryover testing (blank injections immediately after the 1000 ng/mL calibrator) showed no residual signal in chromatograms or EICs. Calibration across 20–1000 ng/mL (20, 40, 200, 500, 800, 1000 ng/mL) over five days in aged bovine blood fit a quadratic model with R^2^ > 0.99 for all analytes, satisfying SWGTOX criteria. The limit of detection (LOD)/limit of quantification (LOQ) were set at 20 ng/mL (the lowest non-zero calibrator) based on toxicological relevance and were verified using 15 replicates, meeting a %CV ≤ 20%; this threshold is forensically/clinically appropriate for amphetamine-related drugs in blood. Precision (*n* = 3 at six levels over five days) met SWGTOX (CV ≤ 20%), with intra-day CVs of 1.00–18.30% and inter-day CVs of 6.60–19.70%. Accuracy from blinded QCs met a ±20% bias (−11% to +18.25%), indicating no systematic error. Autosampler stability at 10 °C for 36 h (40, 500, 1000 ng/mL) remained within ±20% of initial responses, supporting hold times without degradation. All metrics met acceptance criteria, supporting fitness for forensic quantification of amphetamine-related drugs, including BMPEA, in whole blood.

The validated UPLC-qTOF-MS analytical method in conjunction with mixed-mode solid-phase extraction (MMSPE) successfully determined the concentrations of β-methylphenethylamine (BMPEA) and identified its metabolites in rat cardiac blood samples. Excellent performance characteristics were shown by the analytical parameters for both BMPEA and the internal standard amphetamine-d11. BMPEA showed a molecular ion [M + H]^+^ at *m*/*z* 136.1121 and key fragment ions at *m*/*z* 119.0855 (quantifier ion) and *m*/*z* 91.0542 (qualifier ion), whereas amphetamine-d11 displayed corresponding ions at *m*/*z* 147.1653, 130.1388, and 102.1075, respectively. With BMPEA eluting at 6.21 min under the specified analytical conditions, the chromatographic separation produced baseline resolution between BMPEA and amphetamine positional isomers. The method’s dependability for quantitative analysis was demonstrated by calibration curves built over the concentration range of 20–1000 ng/mL, which showed quadratic regression behavior with excellent correlation coefficients (R^2^ > 0.99), confirming the method’s reliability across the validated working range. The averaged quadratic calibration equation (y = 0.0003×^2^ + 0.0341x + 0.0149) provided robust quantification capabilities with acceptable precision and accuracy parameters.

**Figure 2:**
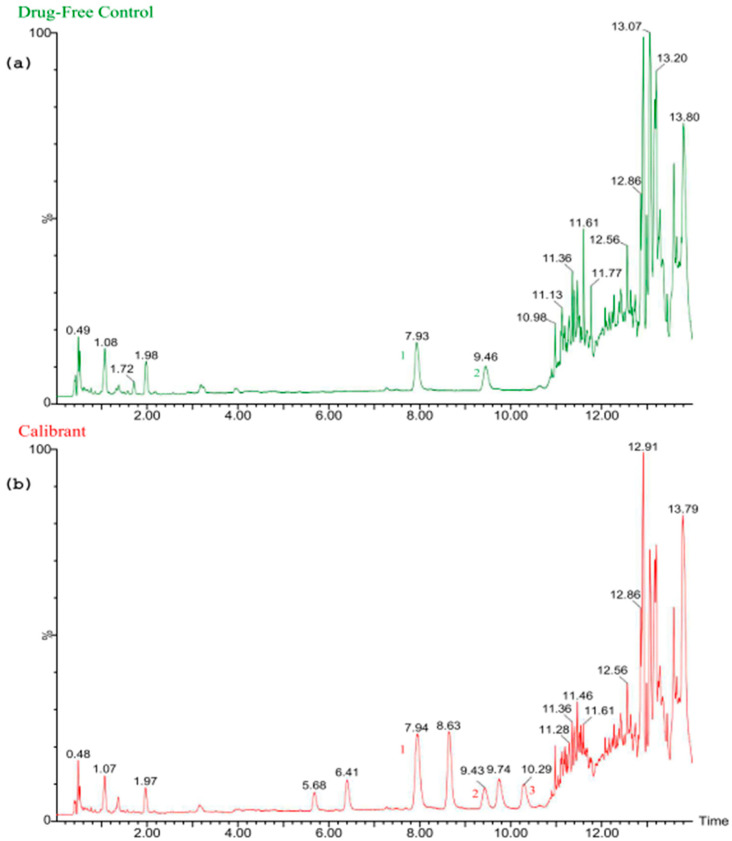
Total ion chromatogram of extracted rat postmortem whole blood: (**a**) drug-free control, and (**b**) sample spiked with 800 ng/mL of the combined working solution and 500 ng/mL of the internal standard solution; (1) ephedrine-d3, (2) amphetamine-d11 and (3) β-methylphenethylamine.

The UPLC-qTOF-MS data for BMPEA in rat drug-free perimortem blood samples prepared by MMSPE are summarized in Table 1 and are shown in Figure 2.

### 3.1. Quantitative Analysis of BMPEA

**Figure 3:**
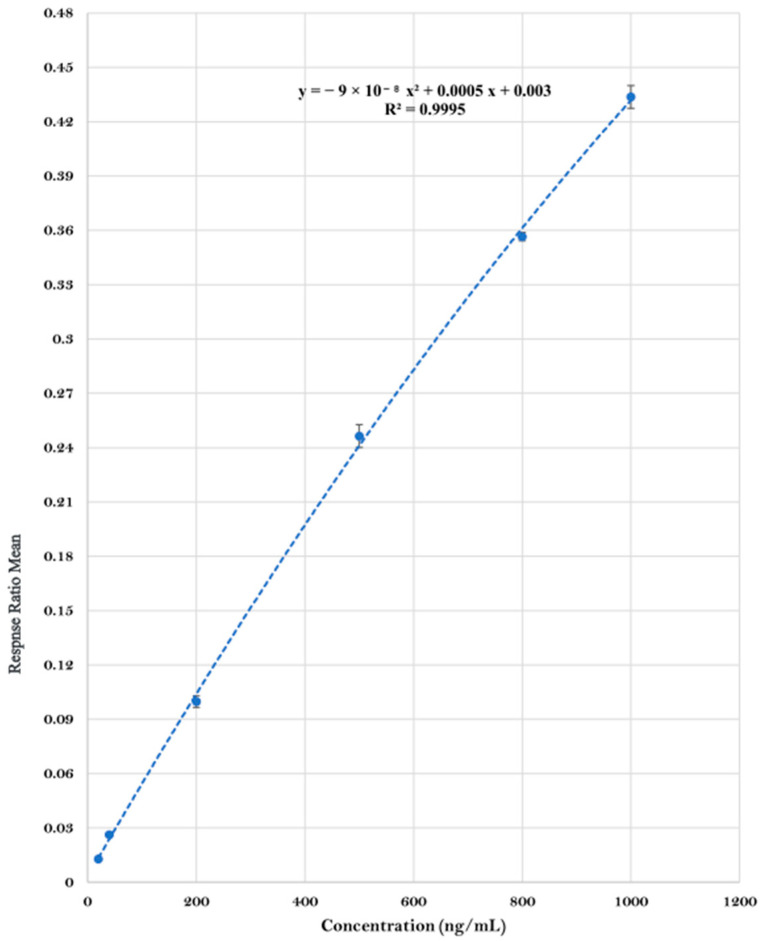
Averaged quadratic calibration curve of β-methylphenethylamine. Error bars represent the standard error of the mean of the response ratio of duplicate samples at each concentration level.

**Figure 4:**
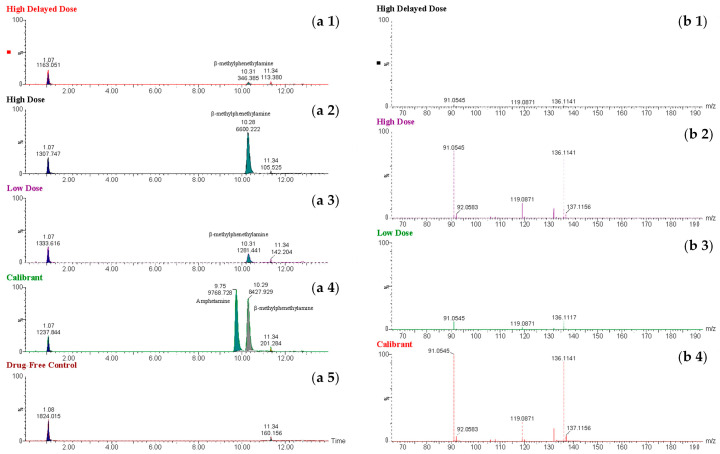
Extracted ion chromatograms (**a1**–**a5**) obtained using the molecular ion *m*/*z* 119 for β-methylphenethylamine from extracts of perimortem whole-blood (rat) samples: (**a1**) high-dose delayed-sampling, (**a2**) high-dose, (**a3**) low-dose; (**a4**) calibrant at a concentration of 1000 ng/mL; (**a5**) drug-free control. Mass spectral profile (**b1**–**b4**) of β-methylphenethylamine in extracts of perimortem whole-blood samples: (**b1**) high-dose delayed-sampling, (**b2**) high-dose, (**b3**) low-dose; (**b4**) calibrant at a concentration of 1000 ng/mL.

BMPEA concentrations in rat cardiac blood were fitted with quadratic regression lines, and concentration dependence was assessed over a 20–1000 ng/mL range. The findings revealed a strong correlation (R^2^ > 0.99). The averaged quadratic calibration curve is shown in Figure 3. The BMPEA concentrations in the rat cardiac blood from the three groups (low, high, and high-dose delayed-sampling groups) were determined using the averaged quadratic regression equation of the two constructed standard curves (Figure 4).

The determined concentrations of BMPEA are shown in Table 2. The high-dose group samples, collected within 20 min post-injection of 30 mg/kg BMPEA, demonstrated the highest mean concentration of 869 ± 29 ng/mL, with individual sample concentrations ranging from 841 to 899 ng/mL. In contrast, the high-dose delayed-sampling group samples, collected 90 min after the same dose administration, exhibited dramatically reduced concentrations with a mean of 31 ± 9 ng/mL and individual values ranging from 22 to 40 ng/mL. The low-dose group, given 10 mg/kg BMPEA, showed correspondingly lower but detectable concentrations, with a mean of 104 ± 25 ng/mL and individual measurements ranging from 85 to 132 ng/mL. All determined concentrations (22–899 ng/mL) across the experimental groups were within the validated analytical range of 20–1000 ng/mL, confirming the method’s applicability for pharmacokinetic studies. The measured concentrations of BMPEA are shown in Table 3.

### 3.2. BMPEA Metabolites Identification Analysis

**Figure 5:**
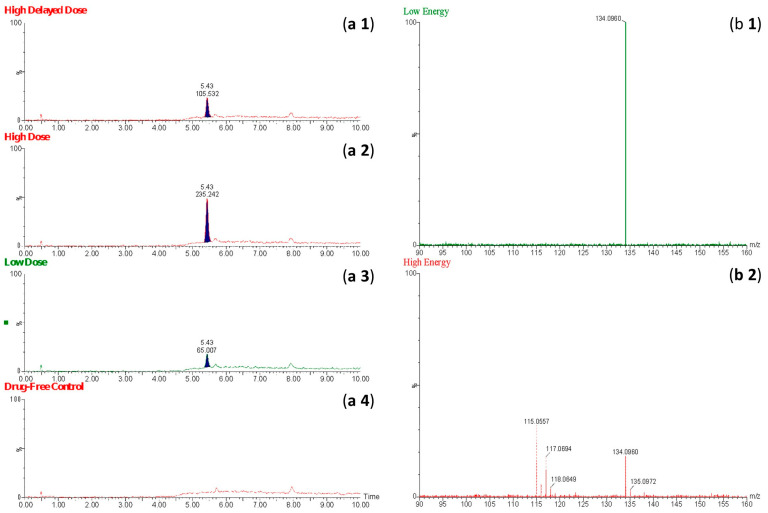
Extracted ion chromatograms (**a1**–**a4**) obtained using the fragmented ion *m*/*z* 134 for β-methylphenethylamine in extracts of perimortem whole-blood samples: (**a1**) high-dose delayed-sampling, (**a2**) high-dose, (**a3**) low-dose; (**a4**) drug-free control. Mass spectra of the proposed metabolite of β-methylphenethylamine obtained at low energy (**b1**) and high energy (**b2**).

**Figure 6:**
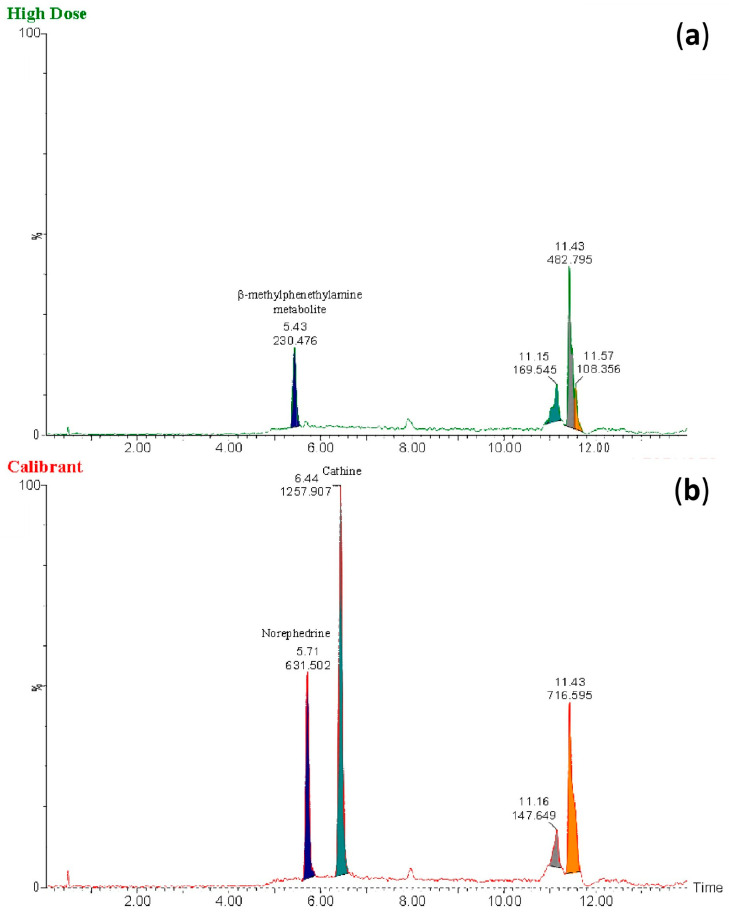
Extracted ion chromatograms obtained using the fragmented ion *m*/*z* 134 for (**a**) the metabolite of β-methylphenethylamine in extracts of the high-dose rat perimortem whole-blood samples and (**b**) norephedrine and cathine at a concentration of 20 ng/mL in the calibrant.

**Figure 7:**
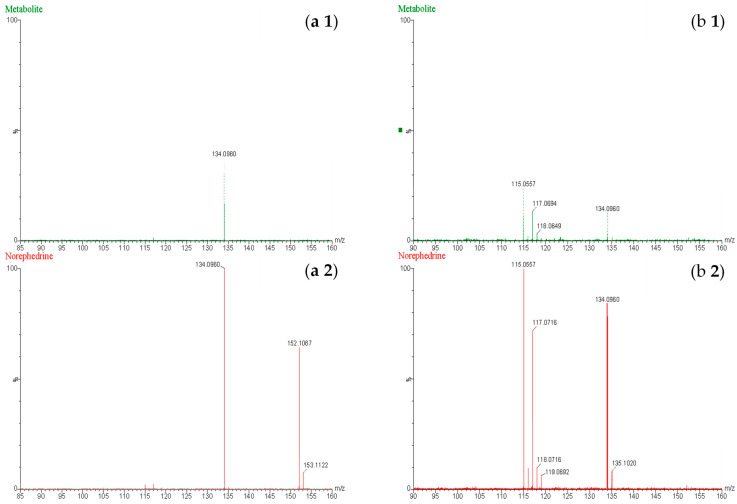
Mass spectra obtained at low energy (**a**) and high energy (**b**) for (**a1**,**b1**) the proposed metabolite of β-methylphenethylamine and (**a2**,**b2**) norephedrine at a concentration of 20 ng/mL.

**Figure 8:**
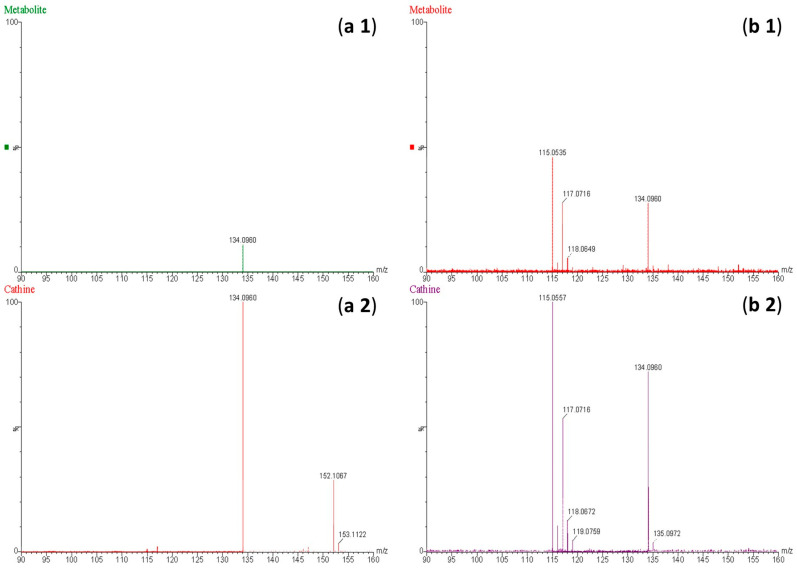
Mass spectra obtained at low energy (**a**) and high energy (**b**) for the proposed metabolite of β-methylphenethylamine (**a1**,**b1**) and cathine (**a2**,**b2**) at a concentration of 20 ng/mL.

Through comprehensive mass spectrometric analysis, metabolite identification analysis identified the presence of a novel BMPEA biotransformation product. Using both low-energy and high-energy acquisition modes, a compound that produced a fragmented ion at *m*/*z* 134 was consistently found in all drug-positive rat blood extracts, but it was not present in drug-free control samples. In comparison to low-dose and delayed high-dose samples, the metabolite showed a greater instrumental response in high-dose group samples, indicating dose-dependent formation. Significant structural similarities between the detected metabolite and these phenylpropanolamine positional isomers were found in the fragmentation patterns, especially in the high-energy mass spectra, when compared to reference standards norephedrine and cathine. Chromatographic resolution in line with positional isomer separation attained for amphetamine and BMPEA was demonstrated by the metabolite’s retention time of 5.43 min as compared to norephedrine’s 5.71 min. Figure 5 shows the EIC and mass spectral profiles of the detectable metabolite. A comparative approach was used to compare EICs and mass spectra of the proposed metabolite (Figure 6) with those of NOR (Figure 7) and CAT (Figure 8).

**Figure 9:**
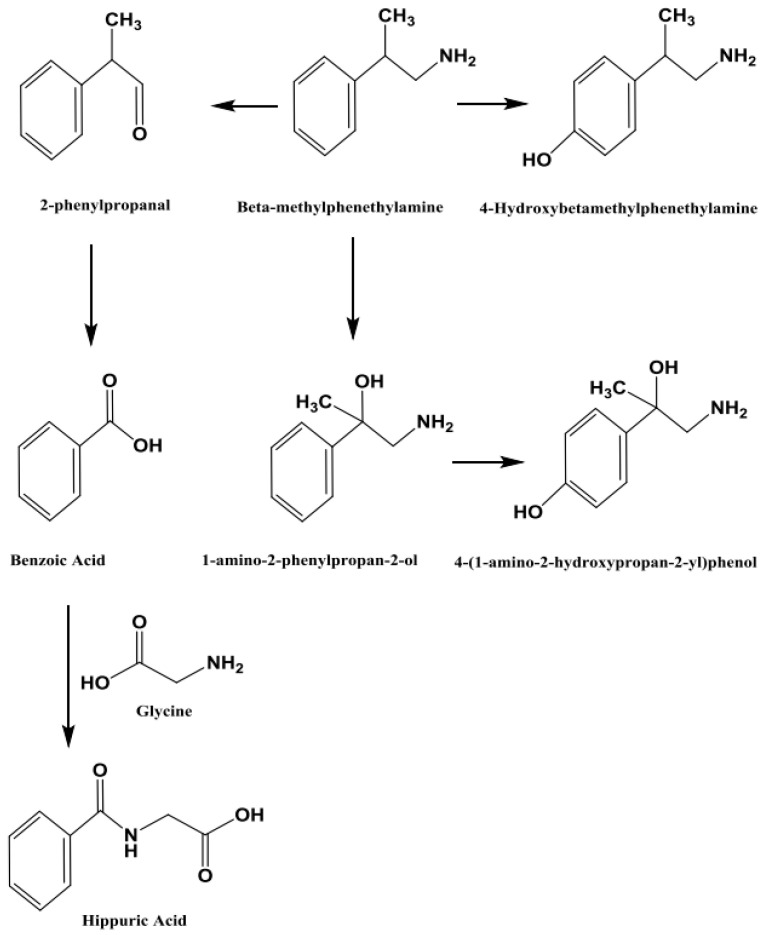
Proposed metabolic pathway of β-methylphenethylamine.

The novel metabolite was determined to be 1-amino-2-phenylpropan-2-ol based on the proposed metabolic pathway that was derived from amphetamine metabolism patterns and validated by mass spectrometric fragmentation analysis, as shown in Figure 9.

**Figure 10:**
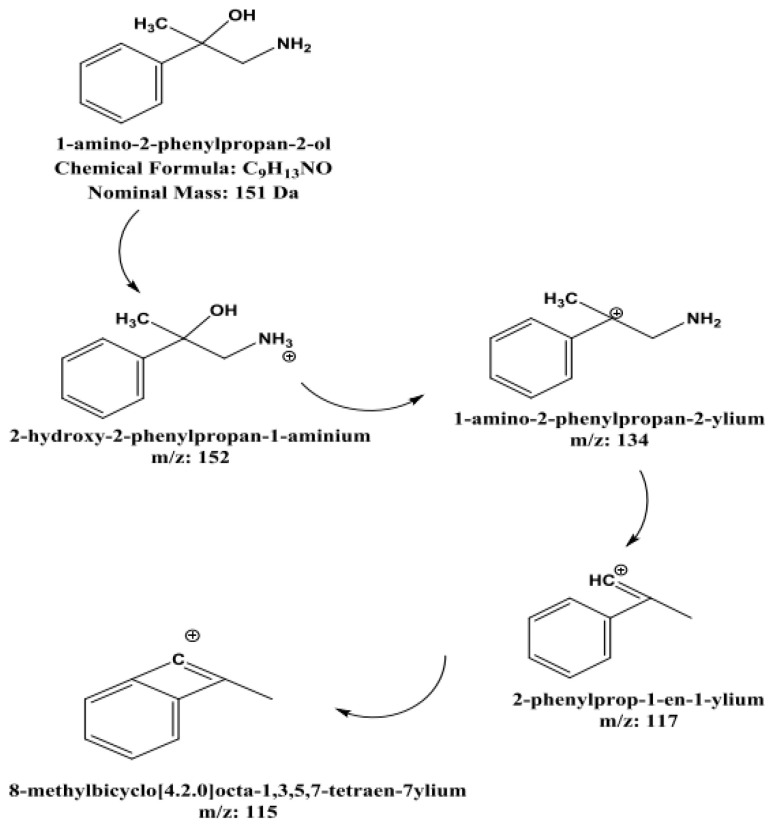
Proposed chemical structures and fragmentation pattern of the metabolite of β-methylphenethylamine.

**Figure 11:**
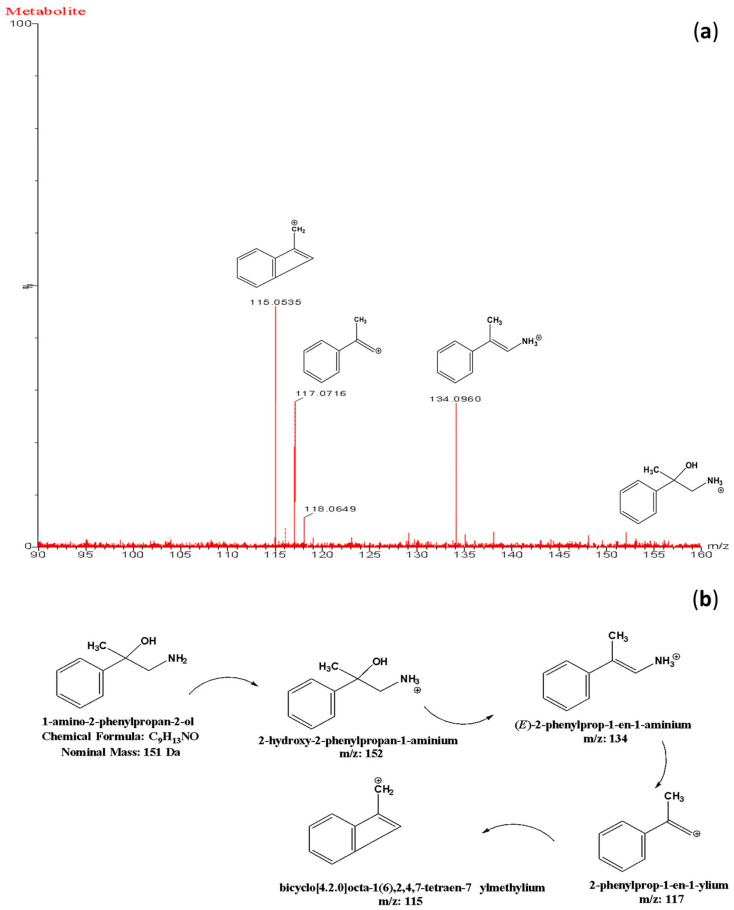
Proposed fragmentation pattern of the β-methylphenethylamine metabolite shown on the high-energy mass spectral profile of β-methylphenethylamine. (**a**) Proposed alternative chemical structures of the β-methylphenethylamine metabolite (**b**).

This metabolite, which is the hydroxylated derivative of BMPEA produced by oxidative metabolism, shows distinctive fragmentation patterns, including water loss (*m*/*z* 134), followed by aromatic stabilization processes. Comparison with theoretical fragmentation patterns and structural analysis further confirmed the identification. However, the molecular ion at *m*/*z* 152 was not detectable, most likely because of in-source fragmentation phenomena related to the XEVO-G2XS qTOF-MS instrument’s StepWave^®^ ion focusing system. Since 1-amino-2-phenylpropan-2-ol has never before been identified as a metabolite of BMPEA, it has greatly advanced our knowledge of the biotransformation pathways of BMPEA and offered promising biomarkers for toxicological and forensic applications. Figure 10 and Figure 11 show the chemical structure of the proposed metabolite and its fragmentation patterns, respectively.

## 4. Discussion

Measurement of *β*-methylphenethylamine (BMPEA), a positional isomer of amphetamine, presents significant analytical challenges due to potential misidentification as amphetamine during routine toxicological screening [9]. This distinction is particularly crucial in clinical and forensic toxicology, especially in cases involving fatal poisoning. Also, BMPEA has been included in the World Anti-Doping Agency’s (WADA) list of prohibited substances [10]. It has been implicated in serious adverse events, notably hemorrhagic stroke, following the consumption of dietary supplements containing this compound [11,12]. Previous pharmacological studies in laboratory rats have demonstrated that BMPEA exhibits properties comparable to amphetamine, with particular concern regarding its potential to produce adverse cardiovascular effects [4]. These emphasize the importance of reliable analytical methods for its detection and discrimination from similar compounds in both clinical and forensic toxicology.

The present study successfully applied and validated the UPLC-qTOF-MS analytical method in conjunction with mixed-mode solid-phase extraction (MMSPE) to determine BMPEA concentrations and identify its metabolites in rat cardiac blood samples, demonstrating the method’s applicability to biological specimens from subjects exposed to BMPEA. Post-injection blood samples from rats were subjected to pretreatment and extracted following established MMSPE pretreatment and extraction protocols [7]. This methodological approach represents a significant advancement in the analysis of BMPEA and its metabolites in complex biological matrices, offering improved sensitivity and specificity. The method’s capability to detect and quantify both the parent compound and its metabolite provides crucial insights into the pharmacokinetic profile of BMPEA in biological systems.

The identification of BMPEA in rat blood was based on agreement between the putative compound and BMPEA (calibrator) in relative intensity at *m*/*z* 136, 119, and 91, and retention time. The EIC (*m*/*z* = 119) and mass spectrum of a calibrant sample (positive control) corresponded to those from a sample derived from a drug-positive rat in terms of the relative intensity of the fragment ions formed and retention times. The S/N ratios were above the lower acceptable limits of 3:1 for *m*/*z* 136 and 91 (qualifiers ions) and 10:1 for *m*/*z* 119 (quantifier ion) in all drug-positive rat blood samples. BMPEA quantification was based on the averaged standard curve using drug-free cardiac rat blood (control group), fit with a quadratic regression equation (R^2^ = 0.9995); Table 1, Figure 3.

The highest determined concentration of BMPEA was 899 ng/mL in a sample obtained from the high-dose group, whereas the lowest determined concentration was 22 ng/mL in a sample collected from the high-dose delayed-sampling group. Both the highest and lowest determined concentrations were within the validated working range of the assay (20–1000 ng/mL). Interestingly, the delayed high-dose samples (collected 90 min post-injection) showed a marked decline in the concentration levels of BMPEA (31 ng/mL ± 9 ng/mL) compared to the high-dose samples (collected within 20 min of injection), which showed very high concentration levels of BMPEA (869 ng/mL ± 29 ng/mL). This finding proved that BMPEA has a short elimination half-life in rat blood. It is consistent with β-phenylethylamine, which exhibits rapid metabolism and a half-life of 6–16 min, depending on the dose [13]. Further experiments are required to precisely estimate the half-life of BMPEA.

The main purpose of this study was to apply the validated method [7] to authentic samples from subjects exposed to BMPEA and to identify one or more BMPEA metabolites. A theoretical metabolic pathway of BMPEA is proposed in Figure 9. This metabolic pathway was proposed based on the published metabolic pathway of amphetamine [14]. According to this theoretical metabolic pathway, 4-hydroxy-β-methylphenethylamine, 1-amino-2-phenylpropan-2-ol, and 4-(1-amino-2-hydroxypropan-2-yl) phenol were proposed to be the metabolites of BMPEA. These proposed metabolites may be expected to be detectable by the analytical method proposed here, whereas 2-phenylpropanol, benzoic acid, and hippuric acid may not provide superior sensitivity and may not be detectable in positive ionization mode [15]. A method for detecting putative metabolites by negative ionization mode was not developed in this work.

Compared with previously developed analytical methods by Piotr C. et al. (2014) [16] and the method used in the study by Alamir A. et al. (2022) [7], significant methodological advances are apparent, particularly in the area of isomer discrimination. The earlier UPLC/MS/MS method was specifically designed to achieve discrimination between BMPEA and amphetamine, offering a targeted approach for these analytes. In contrast, the current UPLC-qTOF-MS approach not only retains the capability to distinguish BMPEA from amphetamine but also extends the analysis to a comprehensive array of isomeric compounds, covering a broad spectrum of amphetamine-related drugs (ARDs) as well as BMPEA and its metabolites. A key enhancement in the current methodology is the adoption of mixed-mode solid-phase extraction, which provides superior cleanup of complex matrices compared with the extraction techniques used in the earlier study. This improved sample preparation, in combination with the high mass accuracy of qTOF-MS and complete baseline resolution achieved through pseudo-isocratic separation, allows for reliable quantification and identification of multiple isomers. Furthermore, the current study includes a stability assessment of analytes for up to 36 h, highlighting its robustness and suitability for forensic applications. Overall, the evolution from the initial UPLC/MS/MS method to the advanced UPLC-qTOF-MS platform demonstrates not only better isomer discrimination but also broader application potential, making it a more versatile tool for toxicological analysis and forensic investigations.

The metabolite identification process was carried out through manual and automated searches. The manual search for molecular ions of common metabolite products (e.g., hydroxylation products) was performed using Masslynx^®^ software. As was observed through analysis of the EICs corresponding to [M + H] (i.e., *m*/*z* = 152; hydroxylated metabolite of BMPEA) or [M + H + 16] (e.g., *m*/*z* = 168; doubly hydroxylated metabolite of BMPEA), no detectable compounds were observed. Considering the fragmentation phenomena of the molecular ions of analytes included in validation within the qTOF-MS used, it is reasonable to anticipate similar patterns with any observed BMPEA metabolites.

The ion focusing system used in the XEVO-G2XS qTOF-MS is known as the StepWave^®^ system (XEVO G2-XS QTof-MS) (Waters, Milford, MA, USA), which plays a significant role in transferring ions from the ion source to the first mass filter (quadrupole) and improving the sensitivity of UPLC-qTOF-MS [17]. The StepWave^®^ uses a relatively high electric field to guide ions toward the first mass filter. Such an electric field may lead to “in-source” fragmentation of certain analytes prior to reaching the mass selector, especially at a low concentration level, leading to low sensitivity of detection of the molecular ion of an analyte. This phenomenon of in-source fragmentation could explain the underlying reason for the inability to detect the molecular ions of the theoretically proposed metabolites. Interestingly, a compound producing the ion with *m*/*z* 134 was detectable in all extracts from drug-positive rats at HE and LE but not in those from the drug-free controls.

Figure 5 shows EICs (*m*/*z* = 134) and mass spectral profiles from extracts of drug-positive and drug-negative rats. The presence of a compound forming this ion in extracts from the drug-positive animals was demonstrated using the automated Metabolite Identification feature of the UNIFI^®^ software (Waters, Manchester, UK). Unfortunately, UNIFI^®^ was not able to conclusively determine the identity of the proposed metabolite, even though the software was able to detect the compound. Furthermore, a search through the scientific libraries of the UNIFI^®^ software yielded more than 100 candidate compounds. Most of these candidate compounds were excluded based on their chemical structures and compositions (chemical formula and nominal mass).

The current study predicted BMPEA metabolic pathway. 4-hydroxy-β-methylphenethylamine, 1-amino-2-phenylpropan-2-ol, and 4-(1-amino-2-hydroxypropan-2-yl) phenol were proposed metabolites. In this regard, two candidates underwent comparison with the proposed metabolite at the level of mass spectral profiles. These candidate compounds were 4-hydroxyamphetamine and norephedrine. Since norephedrine was included in the validated method, its EIC and mass spectra at HE and LE were compared with those of the detectable metabolite, as shown in Figure 7 and Figure 8. There was agreement between the spectra of norephedrine and the proposed metabolite in the HE and LE mass spectra (Figure 7a,b). However, the ion with *m*/*z* 152 was not detectable in the mass spectrum of the proposed metabolite, probably due to in-source fragmentation, as suggested earlier. Surprisingly, the HE mass spectra of cathine and the proposed metabolite were in good agreement, as shown in Figure 8b. These findings support the idea that the proposed metabolite might be a positional isomer of phenylpropanolamine (norephedrine and cathine).

Experimentally, this proposition was strengthened by comparison of the retention times of the proposed metabolite, which were 5.43 and 5.71 min, respectively, as shown in Figure 6. This degree of resolution is consistent with that of the positional isomers included in the validated method (i.e., amphetamine and BMPEA). Accordingly, the metabolite of BMPEA was proposed to be 1-amino-2-phenylpropan-2-ol which is the corresponding positional isomer of norephedrine in the proposed metabolic pathway of BMPEA (Figure 9). This was one newly detected metabolite of BMPEA, which is proposed to be 1-amino-2-phenylpropan-2-ol. This proposed metabolite structure was determined through its fragmentation pattern by using the MSE acquisition mode of qTOF-MS. Two fragmentation patterns were proposed for 1-amino-2-phenylpropan-2-ol, as shown in Figure 10 and Figure 11.

The formation of this metabolite can provide critical insights into BMPEA’s metabolic fate in the human body. Similar to BMPEA, the presence of 1-amino-2-phenylpropan-2-ol in biological samples such as blood, urine, or tissues can serve as a marker of BMPEA ingestion or exposure. Its detection can aid in confirming exposure to BMPEA in cases of suspected intoxication or overdose and enhances our understanding of BMPEA biotransformation. These insights are pivotal for both clinical diagnostics and the forensic investigation of poisoning cases. The current analytical method markedly improves our capacity to accurately quantify such metabolites. This advancement supports more investigations into BMPEA exposure and its related toxicological effects.

Further experiments are required to confirm the proposed chemical structure of this newly detected metabolite of BMPEA: 1-amino-2-phenylpropan-2-ol. Additional studies focused on structural elucidation will be essential to verify its identity and fully understand its role in BMPEA metabolism.

In addition, 4-hydroxy-β-methylphenethylamine could not be excluded as a candidate metabolite of BMPEA. Since a reference standard for 4-hydroxy-β-methylphenethylamine was not available for inclusion in this study, further investigation could not be carried out. Thus, further experiments are required to confirm the identity of the detected metabolite of BMPEA as 1-amino-2-phenylpropan-2-ol or 4-hydroxy-β-methylphenethylamine, or to exclude both candidates. This confirmatory study can be carried out by analyzing neat standards and spiked blood of 1-amino-2-phenylpropan-2-ol and 4-hydroxy-β-methylphenethylamine, and comparing their mass spectra (HE and LE) with those of the metabolite of BMPEA detected in this study.

This study has several limitations. First, the small, male-only cohort (*n* = 3 per dose, Sprague-Dawley) limits power to assess inter-individual and sex differences; observed variability (SD ±25–29 ng/mL) indicates notable between-animal variation, warranting larger groups for robust pharmacokinetic (PK) estimates and range characterization. Second, the two-time-point design (20 and 90 min) precludes robust PK parameters (C_max_, T_max_, t_1/2_, AUC, and clearance) and population PK modeling, despite indicating rapid elimination. Third, metabolite identification relied on comparative MS and retention time matching to related standards (norephedrine, cathine) rather than an authentic 1-amino-2-phenylpropan-2-ol reference, so definitive structural confirmation is pending; similarly, 4-hydroxy-β-methylphenethylamine cannot be excluded. Fourth, exclusive use of positive ESI may have resulted in missed acidic/neutral metabolites (e.g., glucuronides, sulfates, carboxylic acids such as benzoic and hippuric acids). Finally, only blood was analyzed; inclusion of urine and tissues would better capture distribution, metabolism (especially phase II), and excretion. Despite these constraints, this work provides first experimental evidence of BMPEA metabolism and a validated platform for future animal and human studies.

## 5. Conclusions

The validated UPLC-qTOF-MS method, in conjunction MMSPE, was successfully applied in this study to determine BMPEA concentrations and identify its metabolite in rat blood. There was one newly detected metabolite of BMPEA, which is proposed to be 1-amino-2-phenylpropan-2-ol. The proposed metabolite structure was determined through its fragmentation pattern by using the MS^E^ acquisition mode of qTOF-MS. Further experiments are required to confirm the proposed chemical structure of this newly detected metabolite of BMPEA.

## Figures and Tables

**Table 1:** Analytical parameters of β-methylphenethylamine and amphetamine-d_11_.

Analyte	Ionization Mode	Molecular Ion (*m*/*z*)	Fragmented Ion (*m*/*z*) (±0.01)	Retention Time (min) (±0.05)
Amphetamine-d11	Positive	147.1938	98.1000 */130.1653	9.43
β-methylphenethylamine	Positive	136.1219	91.0553/119.0868 *	10.28

* Quantifier ion.

**Table 2:** Regression equation and correlation coefficient of a beta-methylphenethylamine concentration curve in rat perimortem blood.

Analyte	Linear Range (ng/mL)	Regression Equation	R^2^
β-methylphenethylamine	20–1000	$y={-9\;\times \;10}^{-8}C^{2}+0.0005C+0.003$	0.9995

**Table 3:** Concentrations of β-methylphenethylamine in perimortem whole blood (rat, *n* = 9) samples.

Dose	Calculated Concentration (ng/mL)	Average (ng/mL)	STDEV (ng/mL)
Low Dose			
Rat 1	132	104	25
Rat 2	96		
Rat 3	85		
High dose			
Rat 1	868	869	29
Rat 2	841		
Rat 3	899		
High dose and delayed sampling			
Rat 1	40	31	9
Rat 2	22		
Rat 3	32		

## Data Availability

The original contributions presented in this study are included in the article. Further inquiries can be directed to the corresponding authors.
